# Commentary: Allergen and Epitope Targets of Mouse-Specific T Cell Responses in Allergy and Asthma

**DOI:** 10.3389/fimmu.2019.02266

**Published:** 2019-09-20

**Authors:** Tuomas Virtanen, Marja Rytkönen-Nissinen

**Affiliations:** Department of Clinical Microbiology, School of Medicine, Institute of Clinical Medicine, University of Eastern Finland, Kuopio, Finland

**Keywords:** allergenicity, allergen, animal allergen, lipocalin, lipocalin allergen, T cell, T cell recognition, T cell epitope

We read with interest the article on the mouse allergen-specific T cell responses of subjects with asthma or rhinitis sensitized to mouse allergens (1). As the authors write, the subject is important because the specific T cell response to mouse allergens, especially to Mus m 1, has remained uncharacterized until now.

Mus m 1 belongs to the lipocalin family of proteins which contains most of the important mammalian inhalant allergens, such as cow Bos d 2, dog Can f 1, Can f 2, Can f 4 and Can f 6, horse Equ c 1 and Equ c 2, rat Rat n 1, and cat Fel d 4 and Fel d 7 (2, 3). The important exception in the list is the major allergen of cat, Fel d 1, which is a secretoglobin (4). The WHO/IUIS Allergen nomenclature database (http://www.allergen.org) contains one mouse allergen, Mus m 1. It is listed to have two isoforms. The UniProt protein database (https://www.uniprot.org) contains multiple existing and potential proteins highly homologous with Mus m 1. On the other hand, Mus m 1 belongs to the group of lipocalin allergens (Mus m 1, Rat n 1, Fel d 4, Can f 6, and Equ c 1) which show considerable amino acid identities (47–67%) among one another (3). Therefore, it is of special interest that Schulten et al. report that the (homologous immunodominant) peptides that are shared between Mus m 1 and other major urinary proteins (MUPs) are significantly more T cell-reactive than the ones that are not shared (1). They suggest that sequence conservation between MUPs, even from different species, can enhance human T cell responses, presumably due to increased frequency of exposure.

Upon studies regarding the human T cell reactivity to Bos d 2, we speculated that the T cell epitopes of lipocalin allergens may colocalize (5, 6). Thereafter, we have reported that when the amino acid sequences of the lipocalin allergens Equ c 1, Bos d 2, and Can f 1 are aligned their T cell epitopes largely colocalize (2, 3, 7). This is also the case with Bos d 2 and Rat n 1 (8), and it also seems to be the case with Can f 4 (9) and Mus m 1 with other lipocalin allergens (unpublished results). As human endogenous lipocalins can show considerable amino acid identities with lipocalin allergens, up to about 60%, as is the case with dog Can f 1 and human lipocalin-1 (von Ebner's gland protein/tear lipocalin) (10), the sequence conservation or evolutionary relatedness can play a role in the allergenicity of lipocalin allergens (2, 3, 6, 7, 10). Importantly, we have observed that the lipocalin allergens examined are poorly immunogenic in a mouse model (11, 12), weakly antigenic *in vitro* (5, 7, 13, 14), and the T cell epitopes examined are recognized suboptimally by human T cells (15, 16). All these findings point to the concept that lipocalin allergens are not recognized optimally, proposedly, because thymic tolerance due to the sequential similarity of lipocalin allergens with endogenous lipocalins, limits the strength of T cell response to the allergens (2, 3). It is well-documented that weak T cell receptor (TCR)-mediated signaling favors the development of T helper type 2 (Th2) immunity (17).

Taken together, it seems plausible that the T cell reactive regions in lipocalin allergens in general colocalize. Basically, this would result from the evolutionary relatedness of lipocalin proteins which is often observable as amino acid identities of considerable level between them. The conservation of immunodominant regions across lipocalin proteins can have important implications for the allergenic capacity of mammalian lipocalin allergens.

## Author Contributions

All authors listed have made a substantial, direct and intellectual contribution to the work, and approved it for publication.

### Conflict of Interest

The authors declare that the research was conducted in the absence of any commercial or financial relationships that could be construed as a potential conflict of interest.
